# Graph Theoretical Representation of Atomic Asymmetry and Molecular Chirality of Benzenoids in Two-Dimensional Space

**DOI:** 10.1371/journal.pone.0102043

**Published:** 2014-07-17

**Authors:** Tanfeng Zhao, Qingyou Zhang, Hailin Long, Lu Xu

**Affiliations:** 1 Institute of Environmental and Analytical Sciences, College of Chemistry and Chemical Engineering, Henan University, Kaifeng, China; 2 Changchun Institute of Applied Chemistry, Chinese Academy of Sciences, Changchun, China; University of Calgary, Canada

## Abstract

In order to explore atomic asymmetry and molecular chirality in 2D space, benzenoids composed of 3 to 11 hexagons in 2D space were enumerated in our laboratory. These benzenoids are regarded as planar connected polyhexes and have no internal holes; that is, their internal regions are filled with hexagons. The produced dataset was composed of 357,968 benzenoids, including more than 14 million atoms. Rather than simply labeling the huge number of atoms as being either symmetric or asymmetric, this investigation aims at exploring a quantitative graph theoretical descriptor of atomic asymmetry. Based on the particular characteristics in the 2D plane, we suggested the weighted atomic sum as the descriptor of atomic asymmetry. This descriptor is measured by circulating around the molecule going in opposite directions. The investigation demonstrates that the weighted atomic sums are superior to the previously reported quantitative descriptor, atomic sums. The investigation of quantitative descriptors also reveals that the most asymmetric atom is in a structure with a spiral ring with the convex shape going in clockwise direction and concave shape going in anticlockwise direction from the atom. Based on weighted atomic sums, a weighted F index is introduced to quantitatively represent molecular chirality in the plane, rather than merely regarding benzenoids as being either chiral or achiral. By validating with enumerated benzenoids, the results indicate that the weighted F indexes were in accordance with their chiral classification (achiral or chiral) over the whole benzenoids dataset. Furthermore, weighted F indexes were superior to previously available descriptors. Benzenoids possess a variety of shapes and can be extended to practically represent any shape in 2D space—our proposed descriptor has thus the potential to be a general method to represent 2D molecular chirality based on the difference between clockwise and anticlockwise sums around a molecule.

## Introduction

Many molecular properties are dependent on the molecular shape. When some proteins are active, these protein molecules may double over or twist into radically different shapes. Electrophoresis is a technique that separates macromolecules according to their net electrical charge and shape. Methods related to the generation of shape signatures represent molecular shape, and use shape signatures in both ligand-based and receptor-based molecular design [1]. A novel ligand-based virtual screening method combines shape and electrostatic information into a single, unified framework [2].

Consequently, studies on molecular shapes and quantities have been performed [3]–[9]. Generally, the shape of a molecule refers to its surface area in ordinary 3D space. However, some studies on molecules or superstructures are limited to motion on metal surface or in other 2D space, for example, displacements along surfaces of metallic catalysts can be regarded approximately as motions along a plane [10]. Thus, the investigation of molecular shape in 2D space is also necessary, including 2D chirality descriptions.

Since the molecular plane is automatically a mirror plane, a chiral object in 2D space is an achiral object in 3D space [11], that is, the chirality descriptors in 3D space cannot be directly applied to 2D chirality. Therefore, it's necessary to perform the special development of chirality descriptors in 2D space. Several studies on the degree of 2D chirality have been implemented. Buda and Mislow developed a simple method to measure the degree of chirality of a triangle, which is the departure between a pair of enantiomers of triangles [12]. Zabrodsky and Avnir suggested a continuous chirality measure (CCM) to represent the degree of shape chirality. CCM was developed based on the minimal distances that the vertices of a shape must move in order to attain the nearest achiral symmetry point group. CCM was mainly developed for molecules in 3D space, but the degree of chirality can also be applied to the objects in 2D space, if the reflection plane in 3D was replaced by a reflection line in 2D [13].

Randić approached a descriptor of 2D molecular chirality of benzenoids, called F index, which was derived from atomic asymmetry. The asymmetry of an atom, called atomic sum, was derived from the difference between clockwise and anticlockwise binary codes of the atom [14]. Randić etc. also performed some researches of basic theories of binary codes, for example, two theorems were proven concerning the relationships between 2D chiral classification and binary codes of benzenoids [15]–[16]. These codes exhibit four major shortcomings: 1) the degeneracy of atomic sums was serious; 2) the contribution to atomic asymmetry is not related to the distance between two atoms; 3) the degeneracy of the F index was obvious due to the degeneracy of atomic sums; and 4) the atomic sums and F indexes were evaluated by only a few examples. We decided to evaluate their ability to identify 2D chiral benzenoids in an exhaustive set. The results showed that a number of atomic sums were not in accordance with the classification of the atoms (asymmetric atoms or symmetric atoms), some F indexes were not in accordance with the chiral classification of the benzenoids, and some quantitative representations are counterintuitive (see Section results and discussion).

It's a bonus for the molecules in 2D space that atomic asymmetry can be represented by surrounding the whole molecule in different directions (clockwise or anticlockwise) in a plane. In contrast, it is difficult to implement this kind of method in 3D space. If the four shortcomings of Randić's method can be significantly overcome, it should become an indispensable method for the description of atomic asymmetry in 2D space, and a valuable complement to the 2D chemical graph.

Our studies on the degree of chirality of benzenoids tried to exploit a practical method to represent 2D chirality based on the pioneering work of Randić. This investigation aimed at exploring the improved descriptors, in their ability to overcome the observed limitations of the Randić's descriptors, and in their ability to represent the atomic asymmetry from the symmetrical atoms to the most asymmetric atoms, as well as in their ability to represent the molecular chirality from the achiral molecules to the most chiral molecules.

In detail, by introducing distance factor, we improved the method with a weighting scheme based on distances, and suggested weighted atomic sums to represent atomic asymmetry [17]; by summing the contributions of weighted atomic sums, we suggested a weighted F index to represent the degree of molecular chirality [18]. In this paper, the two descriptors are tested with more than 350 thousand benzenoids enumerated in our laboratory with an in-house program.

The shapes of benzenoids can also be regarded as shapes of graphite and graphene, and have potential to approximately represent similar shapes [19].

## Methodologies

**Figure 1:**
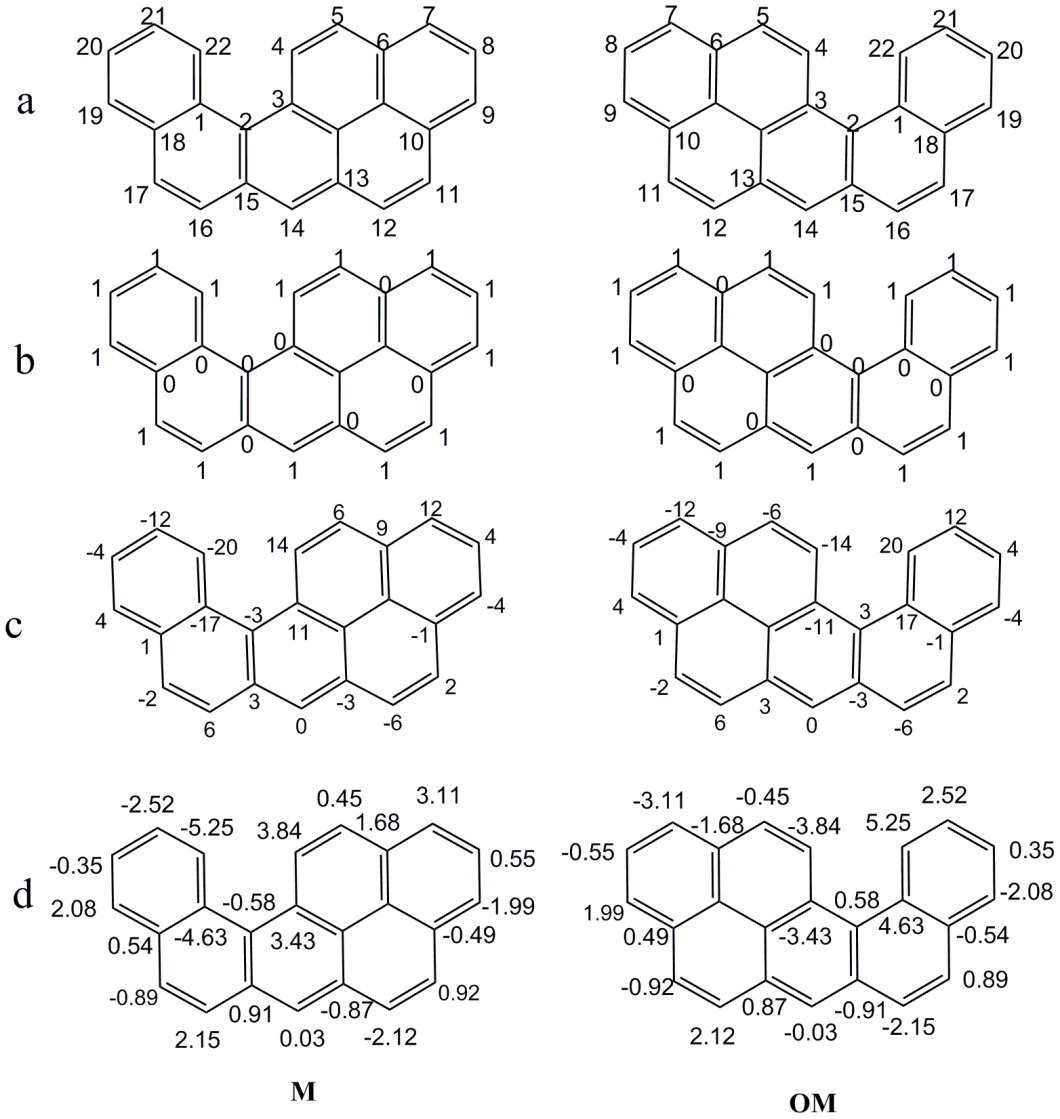
Atomic asymmetry of a pair of enantiomers M and OM. a) atom numbers; b) binary codes; c) atomic sums; d) weighted atomic sums.

In this paper, we limit our interest to 2D space. If no specific declaration is made, the investigation of benzenoids was only performed in the 2D space. If a molecule cannot be translated to its mirror image in a plane as shown in Figure 1, the molecule is chiral in 2D space. Thus, benzenoids can be classified as chiral molecules and achiral molecules in 2D space. We would investigate the qualitative classification and quantitative measurement of molecular chirality.

The descriptors of molecular chirality of benzenoid embedded in a plane were calculated by the following three steps: 1) generation of binary codes of periphery; 2) generation of descriptors of atomic asymmetry; 3) generation of descriptors of molecular chirality.

### 1. Binary codes of periphery

Each benzenoid can be represented by binary codes [20]. As an example, atom numbers and binary codes of a benzenoid M and its enantiomer (OM) in 2D space are shown in Figure 1a and Figure 1b, respectively. The rules to generate the binary codes are briefly introduced as follows:

1. Only the atoms in the periphery are coded. The external vertices of the benzenoid are coded by 0 or 1, and the internal vertexes are neglected.
2. The 1 is assigned to single-ring vertex and 0 is assigned to ring-fusion vertex.

A molecular code can be obtained by gathering the binary codes together as a line notation. Starting from any atom, a clockwise molecular code is obtained by clockwise reading of the binary codes, and the anticlockwise code is obtained by anticlockwise reading of the binary codes. The clockwise molecular code starting with atom 1 of benzenoid M is:
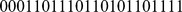



There are 22 atoms along the contour of M, so the code length is 22. The anticlockwise molecular code of M, also starting with atom 1, is:
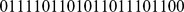



Obviously, the clockwise and anticlockwise molecular codes are different, although starting from the same atom (atom 1).

Also, the anticlockwise molecular code of OM starting with the atom 1 is:
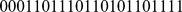



Evidently, the molecular code is the same as the clockwise molecular codes of M. **Thus, characteristic #1 of binary codes can be obtained that the clockwise molecular code starting with an atom in a benzenoid is equivalent to the anticlockwise code starting with the same atom in the mirror of the benzenoid.**

If starting with atom 2, the clockwise molecular code of M is:
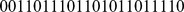



Obviously, this result is different from the clockwise molecular code starting with atom 1. This means that the molecular code depends on the starting atom.

### 2. The representation of atomic asymmetry in two-dimensional space

If the clockwise binary codes of an atom are the same as the anticlockwise binary code of the atom, the atom lies in a symmetric environment and is a symmetric atom. If the clockwise binary code and anticlockwise binary code of an atom are different, the atom is in an asymmetric environment and it is an asymmetric atom. Although a benzenoid can be represented by binary codes, in which binary codes starting from an atom can be used to qualitatively judge the asymmetry of the atom, the binary codes themselves can't be directly used to quantitatively describe atomic asymmetry. Therefore it is necessary to develop quantitative indexes of atomic asymmetry, such as atomic sums suggested by Randić and weighted atomic sums in this paper. An idea suggested by Randić in the literature [14] was also adopted. This idea is that if an atom is asymmetric, the index of its asymmetry should not be zero, which represents the degree of deviation from the symmetry; at the same time; if an atom is symmetric, the index of its asymmetry should be zero.

#### 2.1 Atomic sums

The atomic sums have been suggested by Randić as representation of atomic asymmetry in 2D space [14], and are briefly introduced here. The clockwise (*c*_1_) and anticlockwise (*a*_1_) molecular codes starting from atom 1 of M are listed in Row 2 (count from Table header) and Row 4 of Table 1, respectively, which were used to represent atom 1. Similarly, the clockwise and anticlockwise molecular codes starting from atom 2 can be used to represent atom 2. So can atom 3, and so on.

**Table 1:** The binary codes and partial sums of atom 1 of benzenoid M in Figure 1.

Variable No.(*i*)	0	1	2	3	4	5	6	7	8	9	10	11	12	13	14	15	16	17	18	19	20	21
Clockwise binary codes(c_1_)	0	0	0	1	1	0	1	1	1	0	1	1	0	1	0	1	1	0	1	1	1	1
Clockwise partial sums(*C*_1_)	0	0	0	1	2	2	3	4	5	5	6	7	7	8	8	9	10	10	11	12	13	14
Anticlockwise binary codes(a_1_)	0	1	1	1	1	0	1	1	0	1	0	1	1	0	1	1	1	0	1	1	0	0
Anticlockwise partial sums(*A*_1_)	0	1	2	3	4	4	5	6	6	7	7	8	9	9	10	11	12	12	13	14	14	14
The distance to atom 1 (*d*)	0	1	2	3	4	5	6	7	8	9	10	11	10	9	8	7	6	5	4	3	2	1
*C*_1_- *A*_1_	0	−1	-2	−2	−2	−2	−2	−2	−1	−2	−1	−1	−2	−1	−2	−2	−2	−2	−2	−2	−1	0

The clockwise partial sums of an atom are obtained from clockwise binary codes of the atom by adding at each site all entries preceding that site. The clockwise partial sums of atom *k* (*C_k_*) can be expressed by equation: 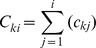



where *C_ki_* is the *i*th element of *C_k_* and *c_kj_* is the *j*th element of clockwise binary codes. For example, the partial sums of atom 1(*C*_1_) are listed in Row 3. Similarly, the anticlockwise partial sums of atom *k* (*A_k_*) are:
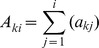



where *A_ki_* is the *i*th element of *A_k_* and *a_kj_* is the *j*th element of anticlockwise binary codes. For example, the partial sums of anticlockwise binary codes (*A*_1_) are listed in Row 5.

In order to observe the difference between the clockwise and the anticlockwise sequences, the anticlockwise partial sums are subtracted from the clockwise partial sums (*C*_1_- *A*_1_), and the sequence of the difference is listed in Row 7.The sum of the entries of Row 7, −34, is the so-called atomic sum. Row 7 (*C*_1_-*A*_1_) in Table 1, **0, −1, −2, −2, −2, −2, −2, −2, −1, −2, −1**, −1, −2, −1, −2, −2, −2, −2, −2, −2, −1, 0, is a vector. It can be found that the number in bold and the number in light is symmetric, thus, atomic sum can be expressed by using half of the vector. As a result, the atomic sum is also the half of the original value. In this paper, the atomic sum of atom *k* is defined as:
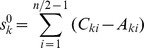



where 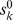 denotes atomic sum of atom *k*; *C_ki_* is variable *i* of *C_k_*; *A_ki_* is variable *i* of *A_k_*; *n* is the number of atoms of contour. If *i*≤n/2, *i* = *d* (distance to atom *k*). Thus, *i* can be replaced by *d* in equation above. 
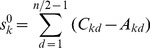
(1)


If the elements of row 7 in Table 1 are put into equation (1), the result is: 
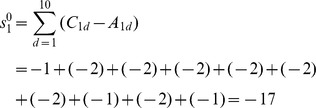



The atomic sum is a measure of the asymmetry of atomic environment of an atom as obtained by circulating around the molecule going in opposite directions (derived from the difference between the clockwise binary codes and anticlockwise binary codes), i.e., −17 represents atomic asymmetry of atom 1 of M. All the atomic sums of M and its enantiomer OM were obtained and are shown in Figure 1c.

For OM, the anticlockwise molecular code starting from atom 1 is: 0001101110110101101111, i.e., this code is just the clockwise molecular codes of M (see before), that is, *C*_1_ of OM will be equal to *A*_1_ of M. Thus, atomic sum of atom 1 of OM will be equal in magnitude and opposite in sign of atomic sum of atom 1 of M, i.e., atomic sum of atom 1 of OM is 17. This feature of atomic sum of atom 1 can be extended to any atom in benzenoids, such as atomic sums of atom 4 of M and OM were 14 and −14. In other words, **the atomic sums of the corresponding atoms of a pair of enantiomers are each other's equal in magnitude and opposite in sign. This is the characteristic #1 of atomic sums.**

**Figure 2:**
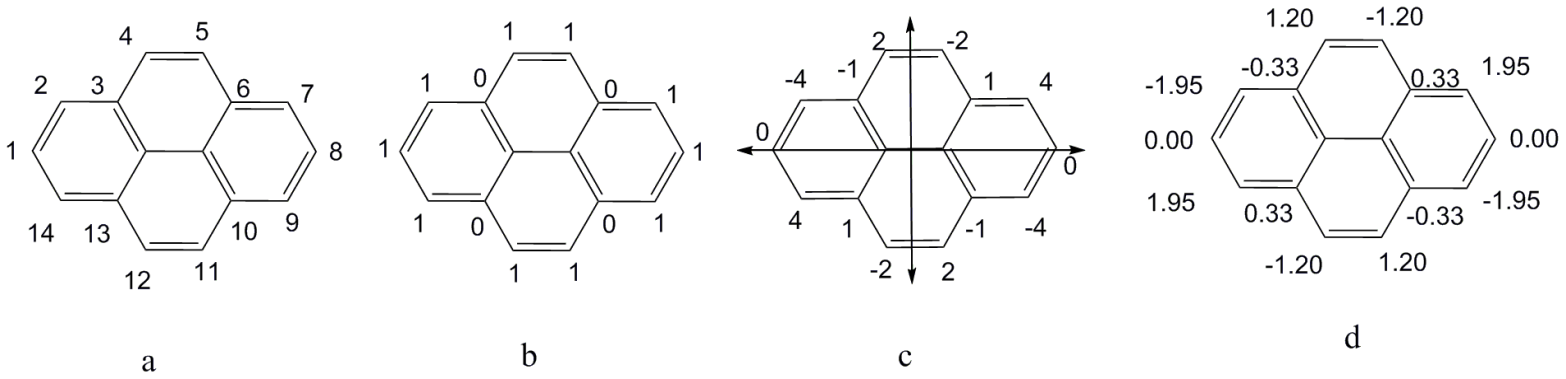
Atomic asymmetry of an achiral benzenoid. The reflection line of molecule was represented by double headed arrow. a) atom numbers; b) binary codes; c) atomic sums; d) weighted atomic sums.

Some planar benzenoids confined to 2D space are chiral, such as the enantiomers in Figure 1; and the others are achiral, such as an anthracene being translated into a benzenoid in 2D space. The reason is that they can be overlapped with its mirror in 2D space. There are self-superimposing reflection lines (symmetric axes) in achiral benzenoids in 2D space as introduced in the literature [16], for example, in the achiral benzenoids of Figure 2 there are two reflection lines represented by double headed arrows. It is obvious that **clockwise binary codes of an atom are the same as the anticlockwise binary codes of its mirror atom about a reflection line in achiral benzenoid, and vice versa. This is the characteristic #2 of binary codes.** For example, atom 4 and atom 5 in Figure 2 possess this characteristic.

Atom numbers, binary codes and atomic sums of an achiral benzenoid with a symmetry axis are displayed in Figure 2a, 2b and 2c, individually. The clockwise binary code of atom 4 is “11011101101110” and anticlockwise binary code is “10111011011101”, i.e., the clockwise and anticlockwise binary codes beginning with the same atom, 4, are different. Thus, atom 4 is an asymmetric atom. It indicates that there are asymmetric atoms in achiral benzenoids. According to equation (1), the atomic sum of atom 4 is 2. It can be found from Figure 2c that atom 4 and atom 5 are symmetric about the vertical double headed arrow and their atomic sums are symmetrical, i.e., 2 and −2. Similarly, this can be extended to all the atoms in achiral benzenoids in 2D space. According to characteristic #2 of binary codes (clockwise binary codes of an atom are the same as the anticlockwise binary codes of its mirror atom about a reflection line in achiral benzenoid, and vice versa) and equation (1), we can get the **characteristic #2 of atomic sum:** **the atomic sum of an atom is equal in magnitude and opposite in sign of its mirror atom about self-superimposing reflection line.**

The clockwise and anticlockwise codes of any symmetric atom are the same, thus, **the atomic sum of any symmetric atom is zero** based on equation (1). **This is characteristic #3 of atomic sums.** As an example of atom 1 in Figure 2 on the horizontal reflection line, both clockwise and anticlockwise binary codes are “11011011101101”, thus atom 1 is a symmetric atom and its atomic sum is zero.

#### 2.2 Weighted atomic sums

On the basis of atomic sums, we have suggested a weighted atomic sum for the representation of atomic asymmetry in 2D space by introducing a distance factor into equation (1). The basic idea of weighted atomic sums is that the farther two atoms are, the less they contribute to the asymmetry of each other. The weighted atomic sum of atom *k* is defined as 
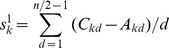
(2)


where 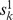 is the weighted atomic sum of atom *k*; *d* denotes the distance (in number of bonds along the molecular contour) to atom *k*; *C_kd_* - *A_kd_* is the difference of clockwise partial sum and anticlockwise partial sum when the distance to atom *k* is *d*; *n* is the number of atoms in the molecular contour. It is emphasized that internal atoms and bonds were not involved in the calculation of atomic sums and weighted atomic sums. i.e., the atomic asymmetry was only derived from the contour of molecular periphery represented by binary codes.

As an example of atom 1 of M, the elements of row 6 and row 7 in Table 1 are put into equation (2), and the result is:
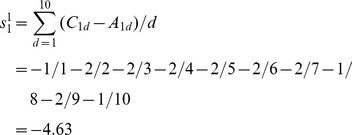



The only difference between weighted atomic sums and atomic sums is the introduction of the distance factor. Thus the distance factor *d* doesn't change the three features of atomic sums mentioned above, that is, the weighted atomic sums as representation of atomic asymmetry in 2D space have the same three characteristics as atomic sums:

1. For a chiral benzenoid, e.g., weighted atomic sum of atom 1 in OM is 4.63 and is equal in magnitude and opposite in sign of the weighted atomic sum (−4.63) of atom 1 in M.
2. For an achiral benzenoid, e.g., the weighted atomic sums of atom 4 and atom 5 are 1.20 and −1.20 in Figure 2d.
3. The weighted atomic sum of a symmetric atom is zero because the clockwise binary codes of a symmetric atom is the same as its anticlockwise binary codes, such as atom 1 in Figure 2d.

### 3. Molecular chirality of benzenoids in 2-dimensional space

#### 3.1 F index

The F index has been suggested by Randić to represent molecular chirality based on atomic sums. The index was obtained by extracting the contributions of atomic asymmetries of the contour of a benzenoid. The F index is defined as a sum of *q*th powers of atomic sums:
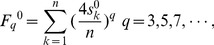
(3)


where, *F_q_^0^* denotes the F index; 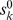 represents atomic sum of atom *k*; *n* is the number of atoms of the contour, which is used to normalize the index; *q* is an odd power, such as 3, 5, 7 (if no specific declaration, the default value of *q* is “3” in this article).

As an example of M, its F index is obtained by putting the atomic sums of Figure 1c into equation (3):
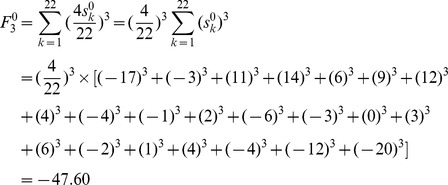



For OM, its F index is:
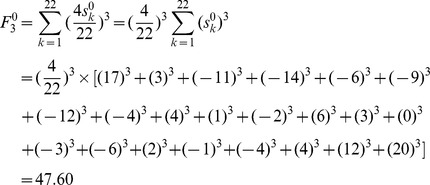



F indexes of **M** and **OM** are equal in magnitude and opposite in sign. Being similar with characteristic #1 of atomic sums, the characteristic of F indexes can be extended to all the pairs of enantiomers. **F indexes of a pair of enantiomers are equal in magnitude and opposite in sign. This is the characteristic #1 of F index.**

As an example of the achiral benzenoid in Figure 2, atomic sums of Figure 2c are put into equation (3), F index is:
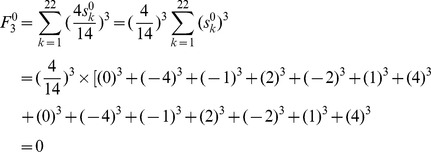



F index is zero, because the atomic sums of two atoms that are symmetric about any reflection line (as the double headed arrow shown in Figure 2) are equal in magnitude and opposite in sign, and each item in equation (3) is canceled by its counter item. It can also be extended to all achiral benzenoids, that is, **F indexes of all achiral benzenoids are zero. This is the characteristic #2 of F index.**

It has been discussed by Randić that if *q* is an even number, F indexes don't satisfy the two characteristics above. Thus, *q* is limited to odd number [14]. In addition, if *q* is equal to 1, F index is zero for each benzenoid. Thus, *q* is not equal to 1.

#### 3.2 Weighted F index

Similarly as atomic asymmetry, the index of a chiral object should not be zero, which represents the degree of deviation from achirality; at the same time, the index of an achiral object should be zero. Due to the low discrimination power, F indexes of some chiral benzenoids are zeros, which means these benzenoids are wrongly classified as achiral. In order to improve the discrimination power of the F index, we defined a weighted F index in which the atomic sums are replaced by weighted atomic sums. The definition of weighted F index is:



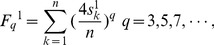
(4)where, 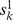
represents the weighted atomic sum of atom *k*; *n* is the number of atoms of the contour; *q* is an odd power and can be 3, 5, 7, and so on. Although weighted F index can be extended to be a vector, we took only a single value (*q* = 3) in this article because a single value of weighted F index already possesses enough discrimination ability. Just like F index, the weighted F index is the sum of the contribution of weighted atomic sums.

Using M as an example, the weighted atomic sums of Figure 1d are put into equation (4), and the weighted F index (*F_3_* ^1^) is: 
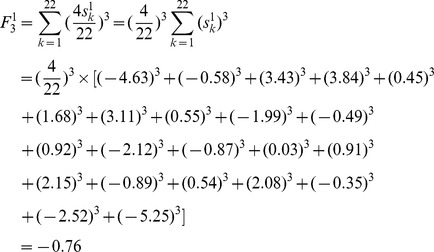



As an example of OM, the weighted atomic sums of Figure 1d are also put into equation (4), and the weighted F index is:
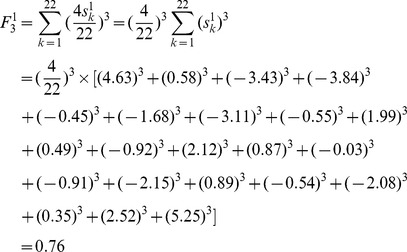



It can be found that, just like the F index, weighted F indexes of M and OM are equal in magnitude and opposite in sign. **Weighted F indexes of a pair of enantiomers are equal in magnitude and opposite in sign. This is characteristic #1 of the weighted F index.**

As an example of the achiral benzenoid in Figure 2, 
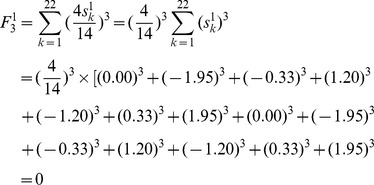



Just like in the F index, each item derived from an atom in equation (4) is canceled by its counter item derived from its mirror atom about self-superimposing reflection line, thus, the **weighted F index of any achiral benzenoid is zero** [14]. **This is the Characteristic #2 of the weighted F index.**

Just like F index, if *q* is an even number, F indexes don't satisfy the two characteristics above. Thus, *q* is limited to odd number in this paper.

### 4. Enumeration of benzenoids in 2D space

**Figure 3:**
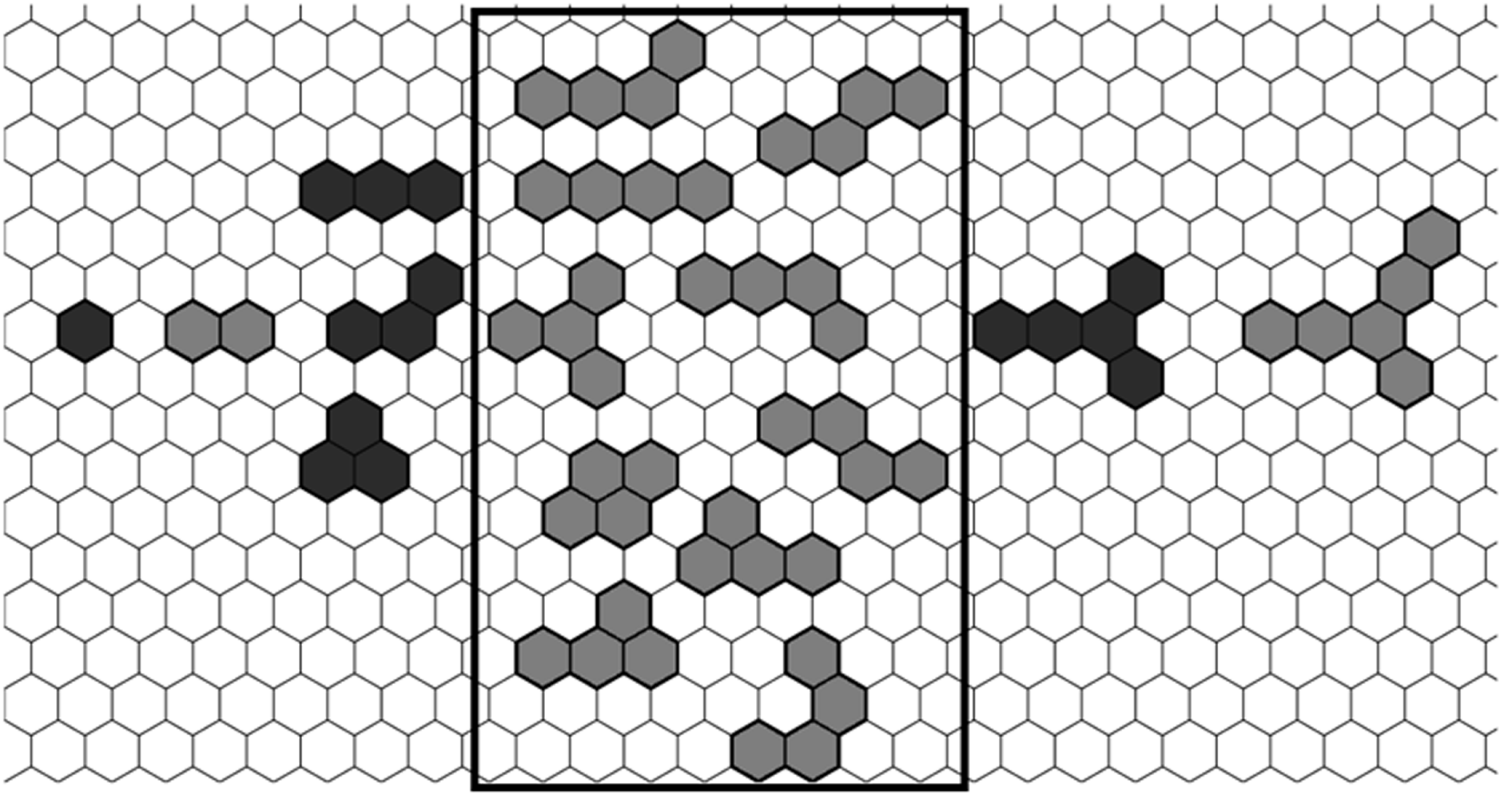
The examples of benzenoids composed of 1–6 benzene rings on a hexagonal lattice.

Some examples of benzenoids are displayed in Figure 3. In this Figure, all the benzenoids composed of 1 to 4 hexagonal rings are displayed, including one benzenoid composed of 1 hexagon, one benzenoid composed of 2 hexagons, three benzenoids composed of 3 hexagons and ten benzenoids composed of 4 hexagons. At the same time, one benzenoid composed of 5 hexagons and one benzenoid composed of 6 hexagons are also shown in Figure 3.

For testing our method, a dataset of benzenoids in 2-dimensional space was obtained by enumeration. The benzenoids in this paper were regarded as planar simply connected polyhexes and all internal regions of benzenoids were filled with hexagons, that is to say, the benzenoids have no internal holes [21]–[22].

**Figure 4:**
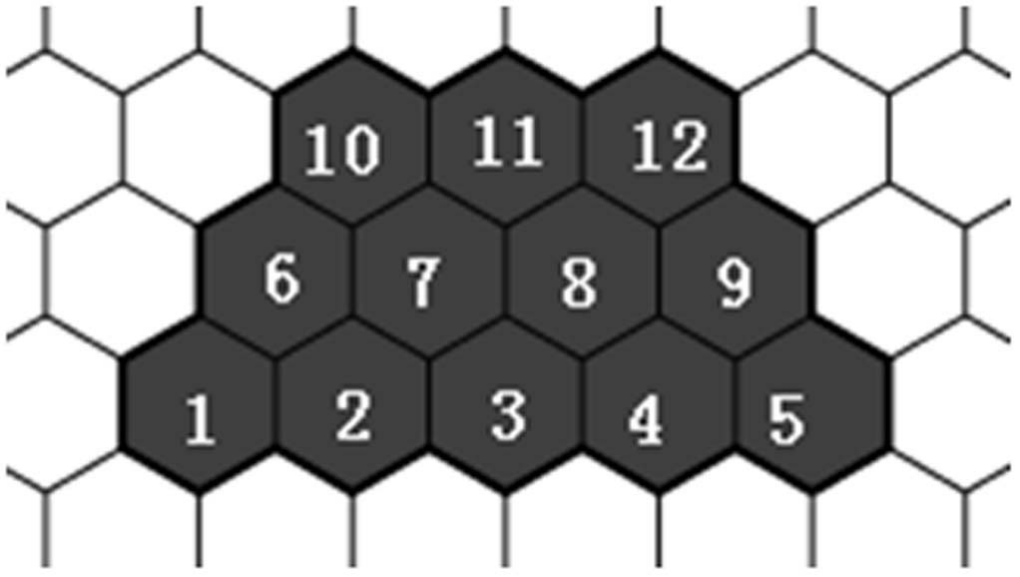
An isosceles trapezoid on a hexagonal lattice.

The enumeration procedure developed in our laboratory is based on the number of given hexagons. For example, if we input 5 as a number of hexagons, the program enumerates all benzenoids composed of 5 hexagons. In order to enumerate all the benzenoids composed of *h* (*h*>2) hexagons, the following procedure were implemented:

Firstly, an isosceles trapezoid was generated. As shown in Figure 4, an isosceles trapezoid region on a hexagonal lattice was used to enumerate benzenoids. Within the isosceles trapezoid region, each hexagon is denoted with a number, and the bases and legs of trapezoid can be easily represented by hexagon number, for example, the legs of trapezoid are represented by {1, 6, 10} and {5, 9, 12}. The length of the leg is the number of hexagons to be contained in a leg, i.e., the leg of trapezoid in Figure 4 is 3.

For the enumeration of benzenoids composed of *h* hexagons, our investigations indicated that the suitable length of each leg (*m*) of the trapezoid is:
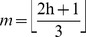
(5)


where, *h* is the number of hexagons of each enumerated benzenoid; *m* is the integer part of the quotient of (2*h*+1)/3. The lengths of the two bases are *h* and *h*-*m*+1. It is proven that this type of isosceles trapezoid in fact contains all of the benzenoids of size *h* in File S1. An example of the isosceles trapezoid drawn for benzenoids composed of *h* = 5 hexagons is shown in Figure 4. The lengths of both legs are *m* = 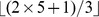 = 3, and the two bases are *h* = 5 and *h*-*m*+1 = 5−3+1 = 3. All the benzenoids composed of *h* = 5 hexagons can be obtained by enumerating benzenoids in such a region.

Each enumerated benzenoid can be represented by the combination of 5 hexagons in terms of hexagon numbers. Hence, the benzenoids composed of 5 hexagons can be represented by {1, 2, 3, 4, 5}, {1, 2, 3, 4, 6}, {1, 2, 3, 4, 7} {1, 2, 3, 4, 8}, and so on.

If benzenoids composed of 3 hexagons (*h* = 3) are enumerated, the length of any leg of the corresponding isosceles trapezoid is *m* = 2 based on equation (5) and the lengths of two bases are *h* = 3 and *h*−*m*+1 = 2. If the trapezoid region is represented by {1,2,3,6,7} in Figure 4, all the three benzenoids composed of 3 hexagons can be obtained, and they are benzenoids {1,2,3}, {1,2,6} and {1,2,7}.

The binary codes were derived from the atoms in the contour of benzenoids. In order to implement this, the program only considers the bonds on the periphery. If a bond only belongs to one benzenoid, the bond is on the periphery. If a bond belongs to two benzenoids, the bond is not on the periphery. No bond belongs to more than two benzenoids. This program was written using the C programming language in our laboratory.

## Dataset

The benzenoids composed of 3 to 11 hexagons were enumerated by the method described in Methodology. The numbers of the achiral benzenoids and all the benzenoids in 2D, as well as all the benzenoids in 3D space are listed in Table 2. 2D achiral benzenoids can be identified by binary codes. It had been proved that a benzenoid is achiral if and only if its binary codes are cp-palindromic. If there are vertices *i* and *j* in a benzenoid, and clockwise binary codes starting with *i* is the same as the anticlockwise binary codes starting with *j*, the binary codes of the benzenoid is cp-palindromic [16].

**Table 2:** The numbers of the achiral benzenoids and all the benzenoids in 2D space, and the number of benzenoids in 3D space.

The number of hexagons	The number of benzenoids in 2D	The number of achiral benzenoids in 2D	The number of benzenoids in 3D
3	3	3	3
4	10	4	7
5	33	11	22
6	146	16	81
7	618	44	331
8	2,803	67	1,435
9	12,824	186	6,505
10	59,883	289	30,086
11	281,648	810	141,229
Summary	357,968	1,430	179,699

From Table 2, it can be found that in 2D space three benzenoids composed of 3 hexagons (all of them are achiral), and ten benzenoids composed of 4 hexagons (four of them are achiral), etc. were obtained. In summary, 357,968 benzenoids in 2D were enumerated, including 1,430 achiral benzenoids. The enumerated results in 3D agreed with that published in the literatures [23].

The chiral benzenoids in 2D space are not chiral molecules in 3D space, i.e., a pair of enantiomers in 2D space become an achiral benzenoid in 3D. For example, M and OM are two chiral benzenoids in 2D space, but they become an achiral benzenoid in 3D space, because OM becomes the same as M when OM is rotated 180° about an axis in the molecular plane. An achiral benzenoid in 2D is still an achiral benzenoid in 3D. Thus, if the number of benzenoids in 2D space is denoted by *N_2D_*, the number of benzenoids in 3D is denoted by *N_3D_* and the number of achiral benzenoids in 2D is denoted by *N_2Da_*, then they satisfy the equation: 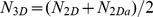
(6)


The numbers of benzenoids in 2D space can't be retrieved from the literature, but the correctness of these numbers can be proved indirectly, because *N_3D_* can be calculated with *N_2D_* and *N_2Da_* based on equation (6). If one or some benzenoids in 2D space was missed, the calculated *N_3D_* must be lower than *N_3D_* in the literature. It can be found that all calculated *N_3D_* by equation (6) are in accordance with *N_3D_* in the literature, and it means that no benzenoid in 2D space was missed, i.e., all the 2D benzenoids were enumerated.

The binary codes of all the enumerated benzenoids are listed in File S2 and each binary code are followed by the positions of the corresponding benzene rings in the isosceles trapezoid region denoted by the hexagon numbers.

The molecular chirality was investigated based on the dataset of 357,968 benzenoids composed of 1,430 achiral benzenoids and 357,968–1,430 = 356,538 chiral benzenoids in 2-dimensional space. The ratio of number of achiral benzenoids to number of all the benzenoids in 2D is 1,430/357,968 = 0.4%. In this work we put special emphasis on chiral benzenoids in 2D. In the periphery of these benzenoids, there are 14,635,116 atoms, which would be used to perform the studies on atomic asymmetry.

## Results and Discussion

### 1. Assessment of atomic asymmetry descriptors

As mentioned above, the asymmetry of a symmetric atom should be represented by zero, and the asymmetry of an asymmetric atom should be a nonzero value, which represents the degree of deviation from the symmetry. The rules outlined above would be utilized to assess atomic sums and weighed atomic sums in this paper.

#### 1.1 Assessment of the classification ability of atomic asymmetry descriptors

As mentioned before, the 14,635,116 atoms of benzenoids in the dataset included 14,633,852 asymmetric atoms and 1,264 symmetric atoms. First the atomic sums of all the asymmetric atoms in 356,538 chiral benzenoids and 1,430 achiral benzenoids of the dataset were calculated, and their ability to discriminate asymmetric and symmetric atoms was assessed. The results showed that the atomic sums of 275,022 asymmetric atoms were zeros, i.e., 1.9% of asymmetric atoms (≈1 atom/52 atoms) were not correctly predicted, For example, the atomic sum of asymmetric atom 14 in Figure 1c is zero, which isn't in accordance with its asymmetry. In contrast, the weighted atomic sum of atom 14 in Figure 1c is 0.03.

**Figure 5:**
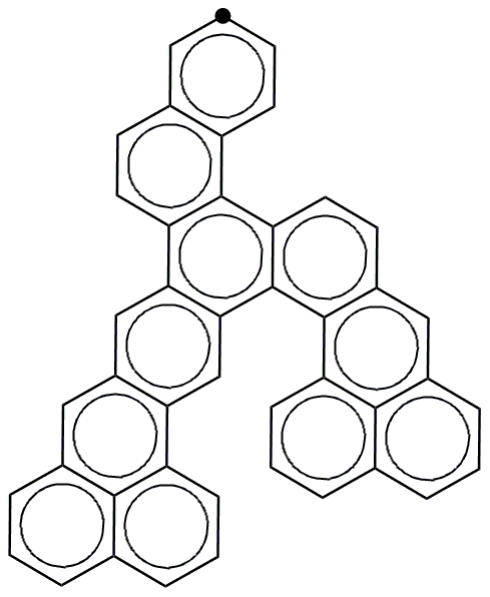
An asymmetric atom possessing weighted atomic sum of zero.

Then the weighted atomic sums of all 14,633,852 asymmetric atoms in the dataset were calculated, and the values of 72 asymmetric atoms were zeros, i.e., <0.0005% asymmetric atoms were wrongly predicted. Thus, weighted atomic sums are satisfactory to classify the atomic asymmetry in 2D. In Figure 5, an asymmetric atom possessing the weighted atomic sum of zero is illustrated, and its calculation is briefly shown as follow. 
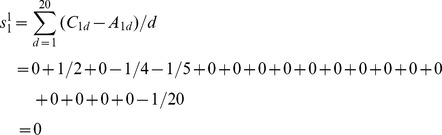



#### 1.2 Studies on quantitative atomic asymmetry

Because the atomic sums and weighted atomic sums of symmetrical atoms are all zeros, thus, only the asymmetric atoms were observed in this section.

It has been observed that many atomic sums were degenerated to the same value with no apparent relationship to chemical intuition, such as the atomic sums of atom 2 and atom 13 of compound **M** were both −3; the atomic sums of atom 9 and atom 20 were both −4; the atomic sums of atom 8 and atom 19 were both 4. But the cases of the weighted atomic sums of these six atoms were quite different from the atomic sums (see Figure 1d), of which, the values for atoms 2, 13 were −0.58, and −0.87; for atoms 9, 20 were −1.99, and −0.35; for atoms 8, 19 were 0.55, and 2.08, respectively. Consequently, weighted atomic sums seem more reasonable than atomic sums to represent the atomic asymmetry of these atoms.

**Figure 6:**
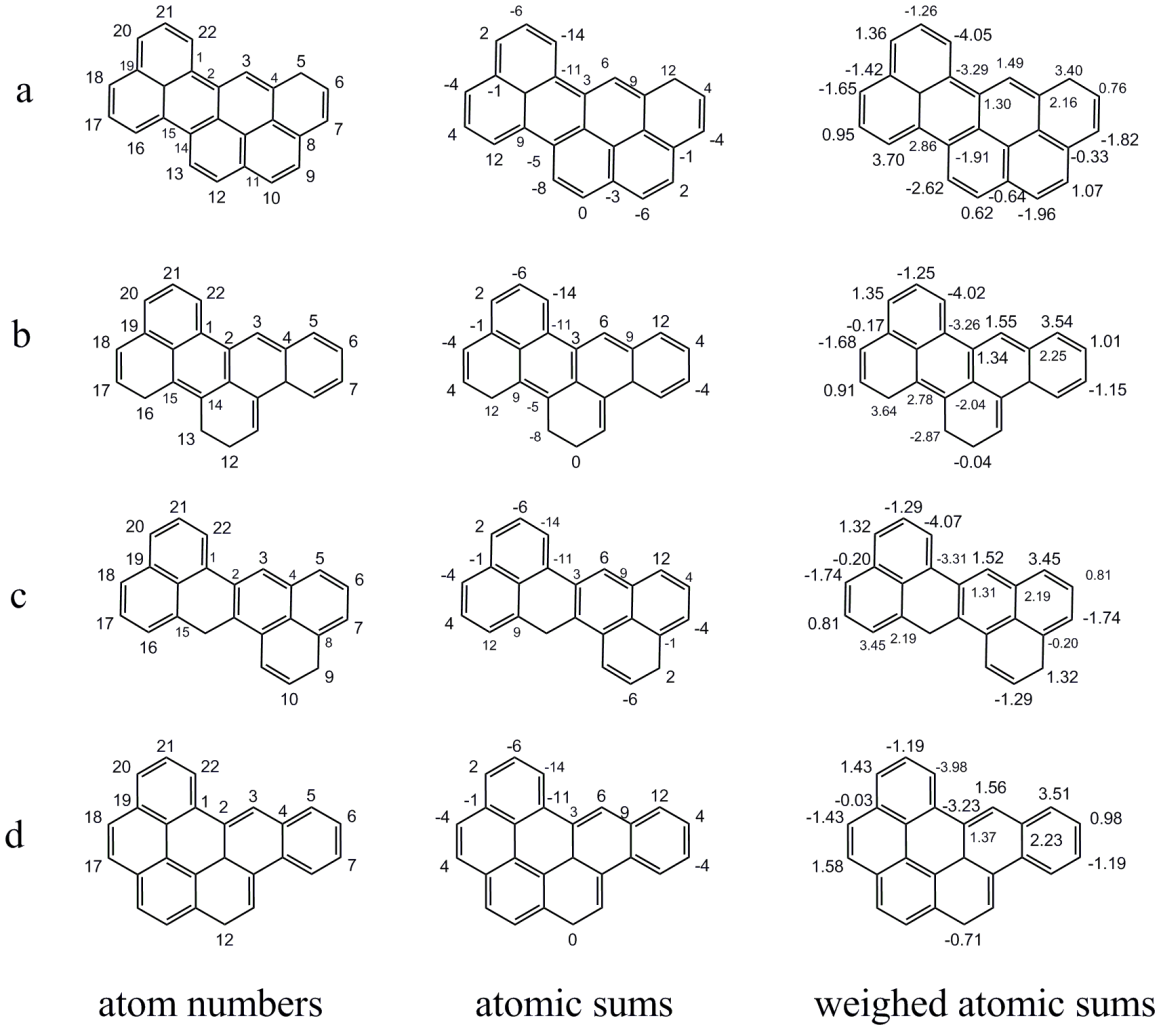
Comparison of sections having the same atomic sums for four benzenoids.

It is also noted that when two similar benzenoids are superimposed, sometimes an overlapped stretch of the two molecules has the same atomic sums. An example of four compounds is given in Figure 6. In which, Figure 6a shows the atom number, atomic sums and weighted atomic sums of all the atoms; Figures 6b, 6c and 6d show the atomic sums that have the same local environment as shown in Figure 6a, although their asymmetric atomic environments were different.

As above, the case of weighted atomic sum was quite different from that of atomic sum. The weighted atomic sums of these atoms are also shown in Figure 6. No two values of weighted atomic sums are the same. Obviously, weighted atomic sums could reduce the degeneracy. The atoms lying in the same local environment have similar weighted atomic sums.

**Figure 7:**
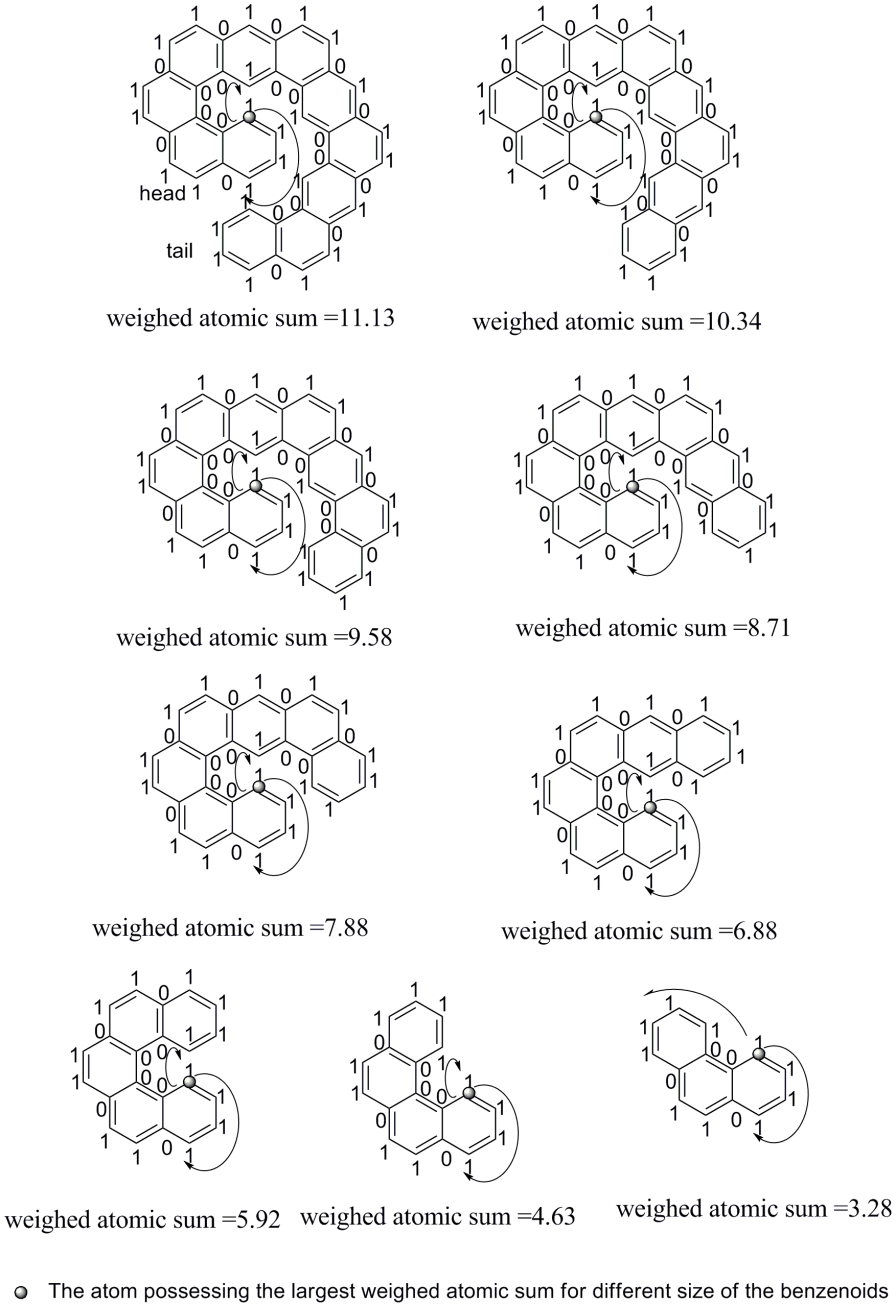
The atoms possessing the largest weighted atomic sums for the benzenoids of sizes 3∼11, separately. The size of a benzenoid is the number of hexagonal rings contained in a benzenoid.

It is interesting to observe the largest weighted atomic sums in different sizes of benzenoids, because this relates with some molecular “shape” (see below). Herein, the size of a benzenoid is the number of hexagonal rings contained in the benzenoid. According to the definition of weighted atomic sum in equation (2), the higher the difference between clockwise partial sums and anticlockwise partial sums of an atom, the larger the weighted atomic sum of the atom. The extreme case of the biggest atomic asymmetry is that all the clockwise binary codes were “1” and all the anticlockwise binary codes were “0”. However, that case could not exist. The atom possessing the largest weighted atomic sum in the dataset is shown on the top left of Figure 7.

In this figure, two arrows starting with the atom possessing the highest weighted atomic sums respectively show the clockwise and anticlockwise directions. The shape looks convex in the clockwise binary codes; and the shape looks concave in the anticlockwise binary codes as shown on the top of Figure 7. The shape of the benzenoid is a spiral ring. In this research, the end including the atom possessing the largest weighted atomic sum is called *head*; and the other end is called *tail*.

The benzenoid above stated contains 11 benzene rings. Like that, the benzenoids composed of *h* = 10 to 3 benzene rings can be obtained by cutting that benzenoid from the end of the tail, and the atoms possessing the largest weighted atomic sum are all in a spiral ring system. From above results, atomic asymmetry could be observed roughly. If an atom lies in a nearly symmetric environment, the value of its atomic asymmetry might be closed to zero; if an atom lies in an environment, and the clockwise shape is nearly convex as well as the anticlockwise shape is nearly concave, its atomic asymmetry is close to the maximum atomic asymmetry.

### 2. Assessment of descriptors of molecular chirality

#### 2.1 Assessment of the classification ability of descriptors of molecular chirality

The conception has been mentioned previously, i.e., if the descriptor of a chiral benzenoid gives zero value, the benzenoid will be wrongly recognized as an achiral benzenoid, and “degeneracy” is called. F indexes (*q* = 3 as default) of 2,958 out of 356,538 chiral benzenoids were zeros, i.e., 2,958 chiral benzenoids were wrongly classified.

If *q* = 3, 5 or 7, individually, then a 3-dimensional F index was obtained. The discriminatory ability of F indexes was increased, but there were still 530 chiral benzenoids to be zero vectors. However, weighted F indexes (*q* = 3 as default) had no zero value. Therefore, all the chiral benzenoids in the dataset were correctly classified based on weighted F indexes.

#### 2.2 Studies on quantitative molecular chirality

**Figure 8:**
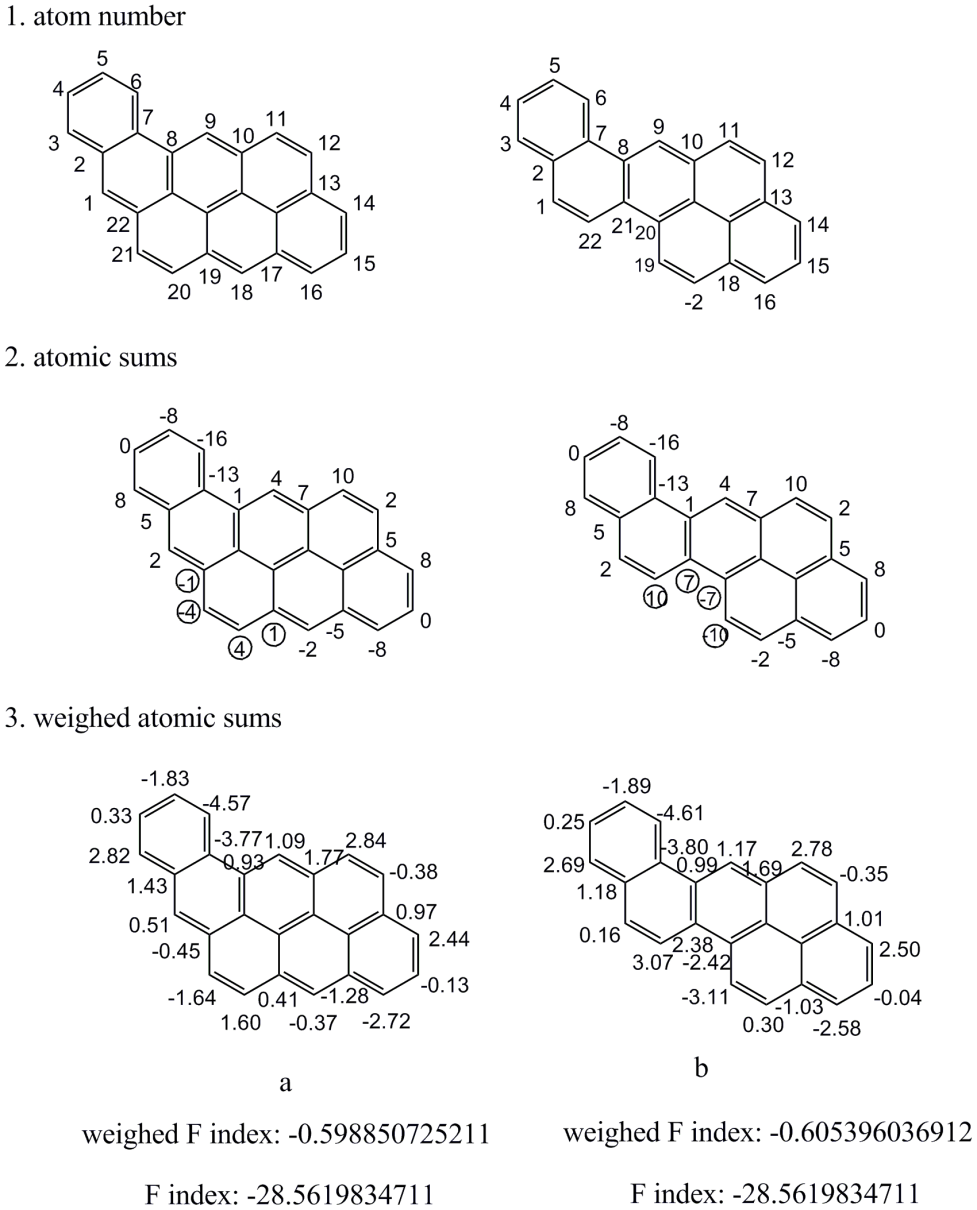
An illustration of two benzenoids possessing the same F indexes vector.

**Figure 9:**
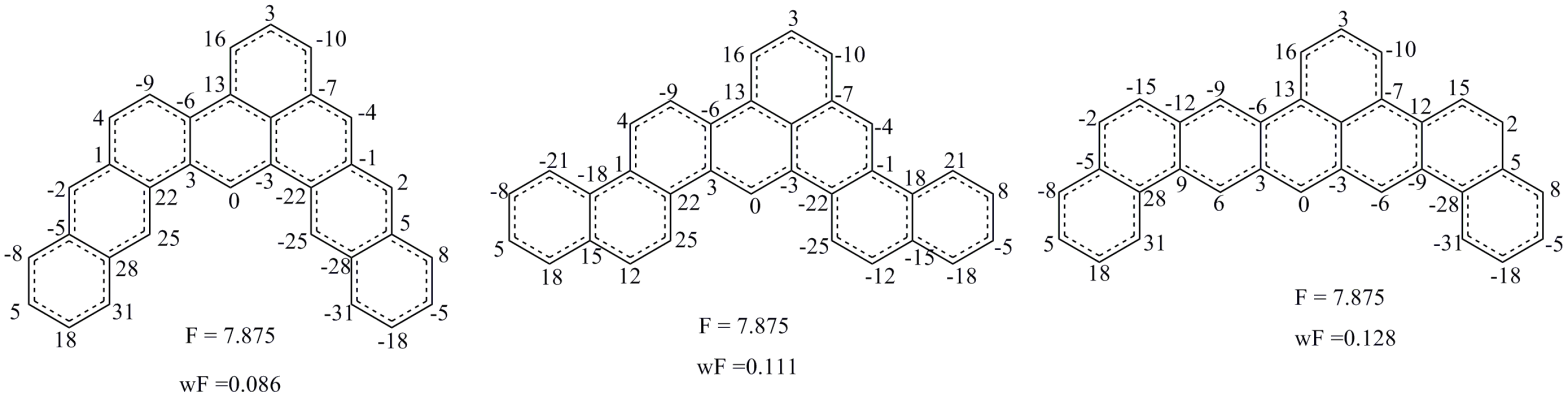
Three benzenoids composed of 8 benzene rings possessing the same F indexes vector.

Some benzenoids possess the same F indexes, such as two benzenoids in Figure 8. For both benzenoids, the contributions of the four circled atomic sums to F indexes are zero values and the remaining atomic sums are the same, which are irrational as discussed in Figure 6. Thus, the ability of F indexes to represent chirality can be put into question. In contrast, the weighted F indexes of the two similar benzenoids are close (−0.599 and −0.605). Similarly, three benzenoids composed of eight benzene rings possessing the same F indexes are shown in Figure 9.

**Figure 10:**
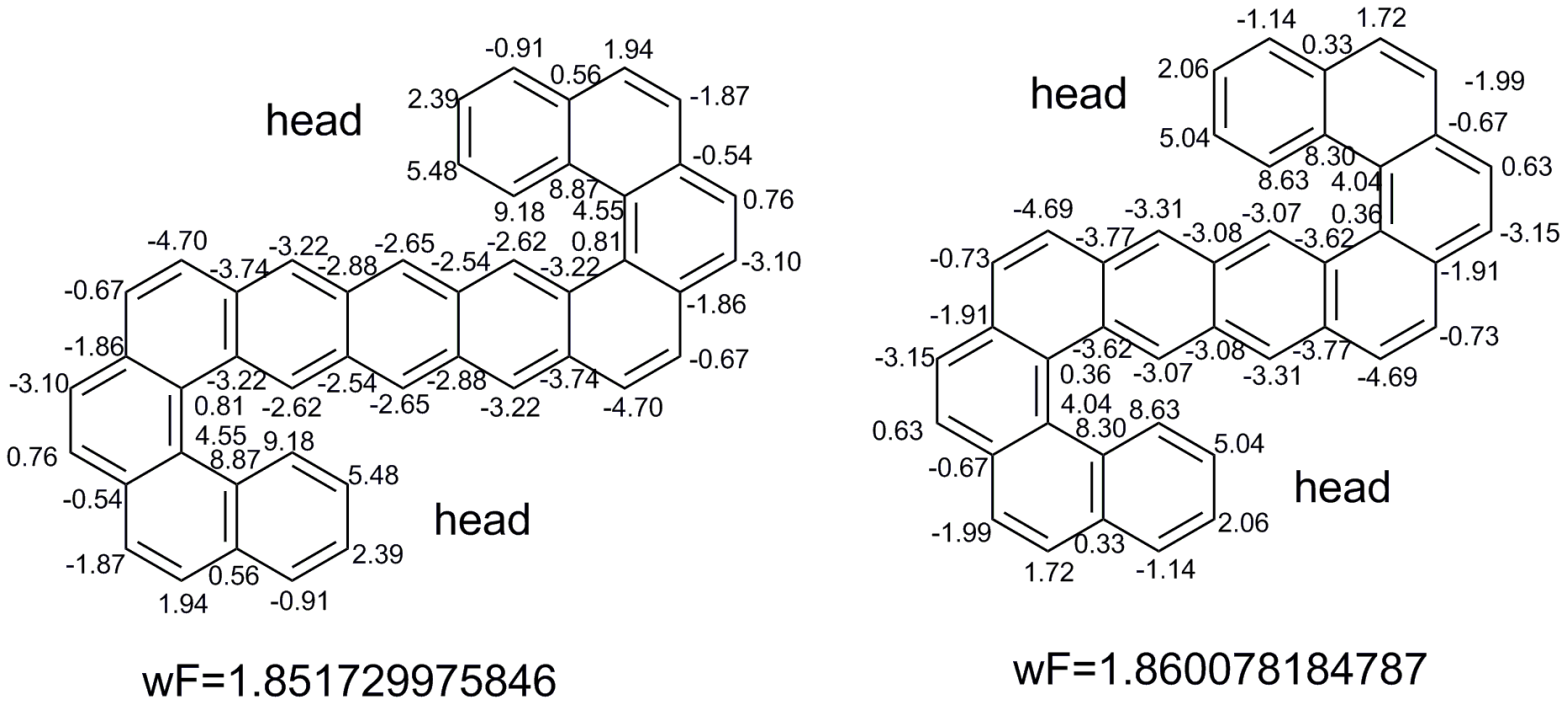
The benzenoids possessing the largest weighted F indexes for benzenoids size of 10 and 11. All the weighted atomic sums are listed.

It is interesting to observe largest weighted F index among the benzenoids size of 11 in Figure 10. It has two head ends that are the same as the head end in Figure 7. For benzenoids size of 10, the benzenoid possessing the largest weighed F index also has a two-head structure.

## Conclusion

In this research, the weighted atomic sums were obtained by circulating around the molecule in opposite directions based on the features in 2D space, and the weighted F indexes were derived from weighted atomic sums. The two indexes were tested by a large data set, and the results indicate that the two indexes here presented are superior to atomic sums and F indexes previously available in the literature. It is noted that due to a wide variety of shapes of chiral benzenoids, the weighted atomic sums as quantitative descriptors of asymmetry and weighted F indexes as descriptors of molecular chirality were suggested by authors, rather than merely labeling objects as being asymmetry, symmetry, chiral or achiral.

The description of benzenoids might approximately be extended to represent any shape in 2D space, if the shape is put on a hexagonal lattice.

Although this study focused on pure graph theoretical aspects of objects in 2D space, this research might be used in QSAR work. For example, the weighted atomic sums have potential to be applied to NMR data prediction for two reasons: 1) both weighted atomic sums and NMR data are sensitive to local symmetry; 2) both of them follow the rule: the farther two atoms are, the less they contribute to each other.

## Supporting Information

File S1 **The proof that any benzenoid composed of** ***h*** **benzene rings can be enumerated in a specific isosceles trapezoid.**
(PDF)

File S2 **The isosceles trapezoid regions used to enumerate benzenoids composed of 3 to 11 benzene rings and all the benzenoids that have been enumerated.**
(ZIP)
