# Supplementary material for: Graph Theoretical Representation of Atomic Asymmetry and Molecular Chirality of Benzenoids in Two-Dimensional Space
Source: PLoS One. 2014 Jul 17;9(7):e102043. doi: 10.1371/journal.pone.0102043 (PMC4102468; doi:10.1371/journal.pone.0102043)
Supplement: File S1 — The proof that any benzenoid composed of h benzene rings can be enumerated in a specific isosceles trapezoid. (PDF) [file pone.0102043.s001.pdf]

PROOF

Herein it is proven that any benzenoid composed of h benzene rings can be enumerated in the specific isosceles trapezoid as described in the Section Methodologies. The length of each leg (m) of the trapezoid is:

$$m = \left\lfloor \frac{2h+1}{3} \right\rfloor$$

where, h is the number of hexagons of each enumerated benzenoid; m is the integer part of the quotient of $(2h+1)/3$. The lengths of the two bases are h and h-m+1.

In a hexagonal lattice, a benzenoid of size h can be regarded as planar connected polyhexes, called h continuous hexagons here. The proof based on pure graph theory is introduced as follows.

A coordinate system (x, y) in a hexagonal lattice is shown in Figure 1. In the system, every hexagon in the grid can be represented by a unique coordinate.

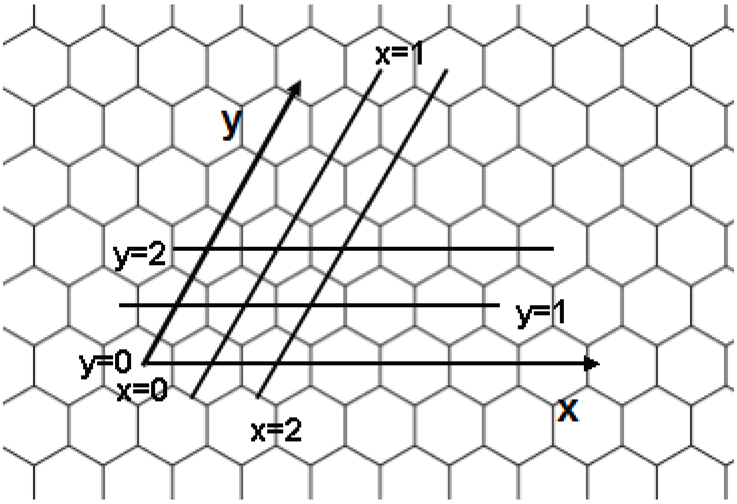

Figure 1. An *xy* coordinate system in a hexagonal lattice

Then a third axis is introduced as shown in Figure 2. Every hexagon still has a unique coordinate. And for every coordinate (x, y, z), it is easy to find that the equation $x + y = z$ is always correct. For example, the coordinates of four grey hexagons in Figure 2 are (0, 0, 0), (1, 1, 2), (1, 2, 3) and (2, 2, 4).

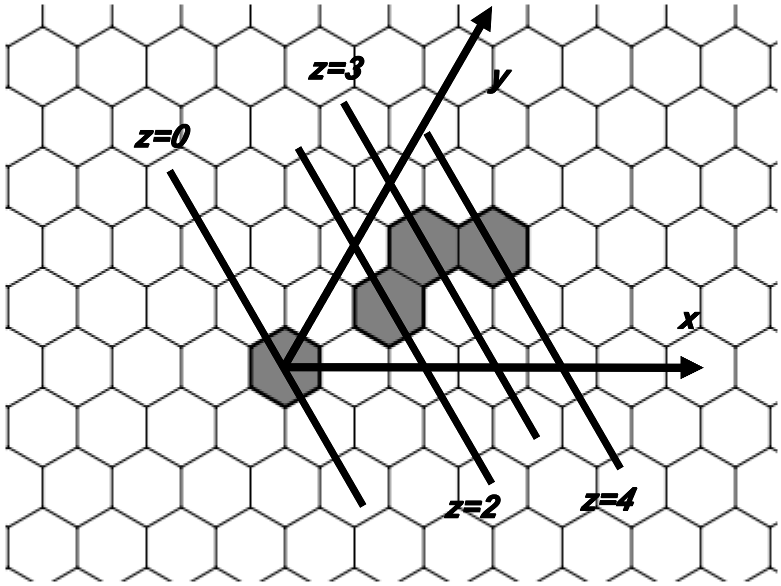

Figure 2. An *xyz* coordinate system with examples of four grey hexagons.

Lemma 1: If h-1 continuous hexagons are added (the hexagon added one by one) from a starting hexagon (x_0, y_0, z_0), z of any hexagon is less than z_0+h.

Proof

Every hexagon has six neighbors next to it, and if the coordinate of a hexagon is (x, y, z), the coordinates of its six neighbors are (x, y+1, z+1), (x+1, y, z+1), (x+1, y-1, z), (x, y-1, z-1), (x-1, y, z-1) and (x-1, y+1, z). In this case, any neighbor (x_N, y_N, z_N) satisfies: $z_N \leq z+1$. Thus, if h-1 continuous hexagons are added one by one from a starting hexagon (x_0, y_0, z_0), z of any hexagon is less than z_0+h.

Lemma 2: If h-1 continuous hexagons are added one by one from a starting hexagon (x_0, y_0, z_0) and the coordinate y of one added hexagon is 0, the value of z of any added hexagon is less than z_0+h-y_0., i.e., $z< z_0+h-y_0$.

Proof

For a hexagon (x, y, z), there are six neighbors as above. The coordinates y of two neighbors decrease, and their coordinates are (x+1, y-1, z) and (x, y-1, z-1). It can be found that the coordinates satisfy: $z_N \leq z$. When the coordinate of starting hexagon is (x_0, y_0, z_0) and the coordinate y of one added hexagon is 0, at least y_0 hexagons satisfying $y_N = y$-1 need be added, that is, at least y_0 hexagons are added, but z doesn't

increase. In this case, if h-1 continuous hexagons are added from the starting hexagon, based on lemma 1 the coordinate of any added hexagon satisfy: z-z_0< h-y_0, that is, the value of z of any added hexagon is less than h-y_0+ z_0., i.e., z<h-y_0+ z_0.

***Lemma* 3: Any benzenoid composed of *h* hexagons can be placed in an equilateral triangular area whose edge consists of *h* hexagons.**

Proof

An equilateral triangular area (the length of any edge is h) on hexagonal lattice is shown in Figure 3 and the coordinate of O is the origin (0, 0, 0). Any benzenoid composed of h hexagons placed on the grid is required to follow two predefined rules:

1) For the coordinate (x, y, z) of any hexagon, there must be $x \geq 0$ and $y \geq 0$;

2) At least one hexagon is placed on x-axis and one hexagon is on y-axis.

It is easy to find that each hexagon on the line DE satisfies the condition z=h-1. In this case, if $z < h$ can be proved for every hexagon (x, y, z) contained in any benzenoid size of h, lemma 3 is proved.

The coordinate of hexagon A is (0, |OA|, |OA|) and the coordinate of hexagon B is (|OB|, 0, |OB|). When hexagon A is regarded as starting hexagon, the remaining h-1 hexagons were added one by one, and z of any added hexagon is less than h-|OA|+|OA| =h based on lemma 2 (z<h-y_0+ z_0). Thus, it is proved that $z < h$.

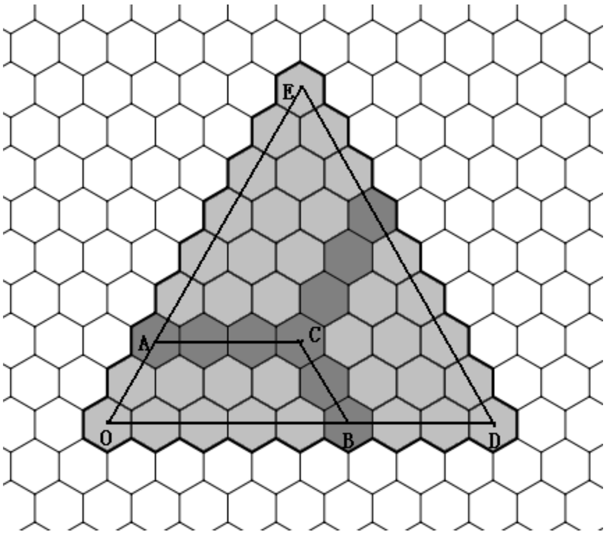

Figure 3. An equilateral triangular area on hexagonal lattice

***Lemma* 4: Any benzenoid composed of *h* benzene rings can be placed in an isosceles trapezoidal area with the parameters: the length of each leg is *m*=$\lfloor (2h+1)/3 \rfloor$; the lengths of the two bases are *h* and *h*-*m*+1.**

Proof

A benzenoid can be rotated in 2D space, and the different poses require different size of trapezoidal area. As an example of a benzenoid shown in Figure 4, there are six ways to place a benzenoid and all the six trapezoidal areas have the same number of hexagons (h) along the lower base, which is in accordance with Lemma 3.

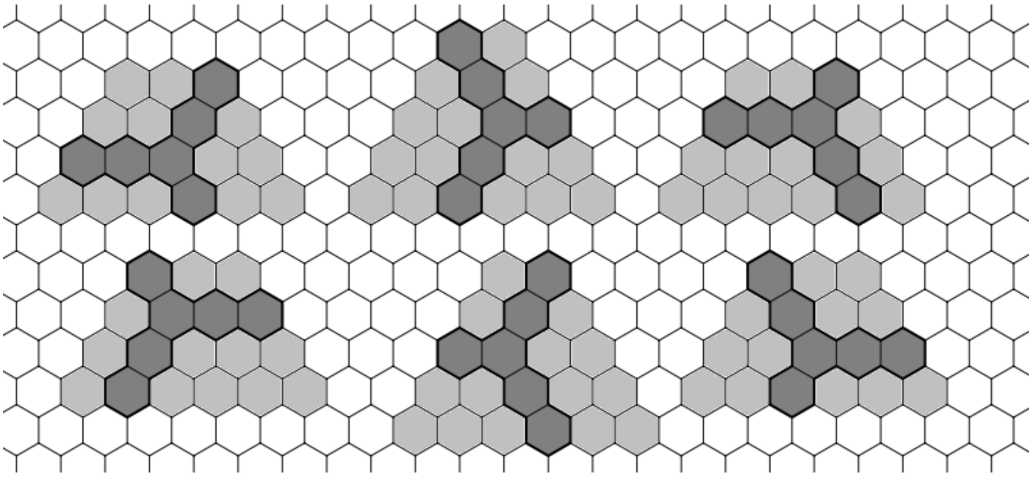

Figure 4. Six poses of a benzenoid and the corresponding trapezoidal area

The ranges of coordinates (x, y, z) of a benzenoid are illustrated in Figure 5. In Figure 5, x_{min} denotes the minimum value of all the x of all the hexagons in a benzenoid, x_{max} denotes the maximum of these x, and y_{max}, y_{min}, z_{max} *and* z_{min} denotes the corresponding maximum and minimum values of y and z. If $X = x_{max} - x_{min}$, $Y = y_{max} - y_{min}$, $Z = z_{max} - z_{min}$, the value of min(X, Y, Z) + 1 is the number of the layers of hexagons in the trapezoidal area are really required.

The benzenoid that looks like a clover has the largest value of min(X, Y, Z) of all the benzenoids with a certain number of hexagons. In order to get this benzenoid a hexagon is added to one of the leaves circling around the central hexagon every time.

The procedure of adding hexagons is shown in Figure 6, and herein $X = \min(X, Y, Z)$.

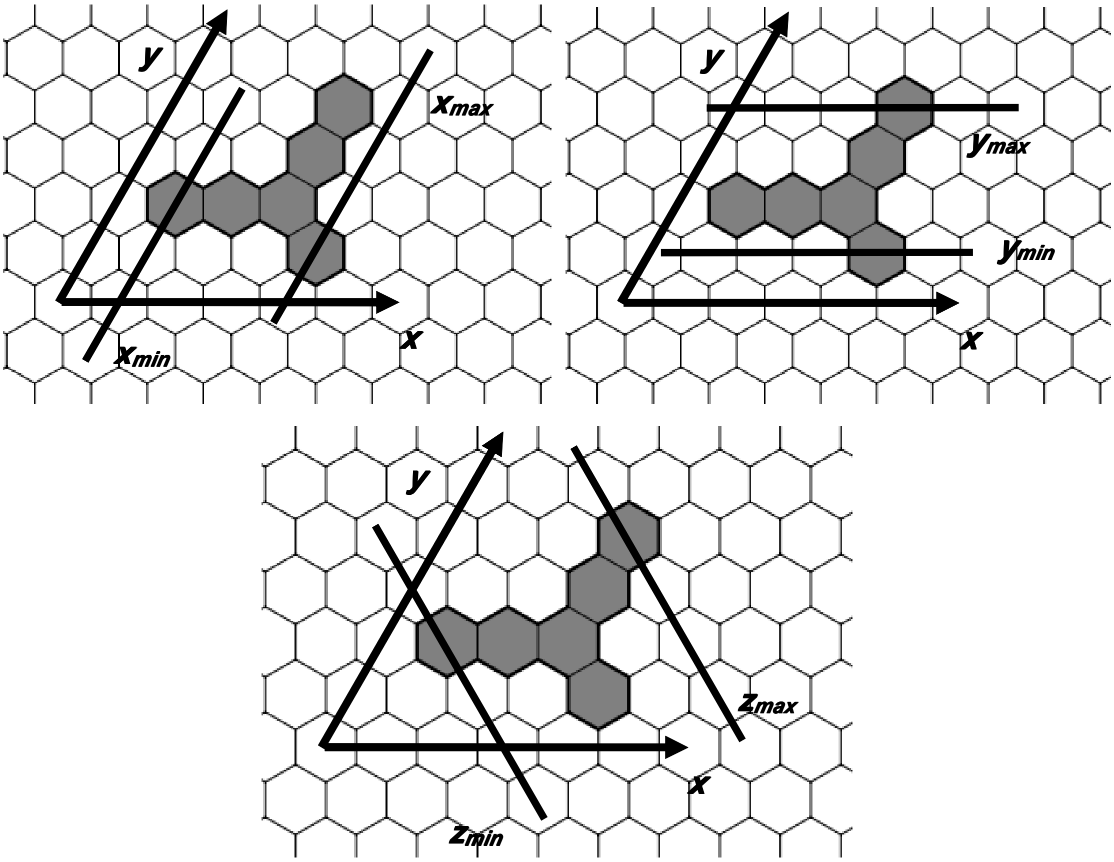

Figure 5. The ranges of coordinates of a benzenoid

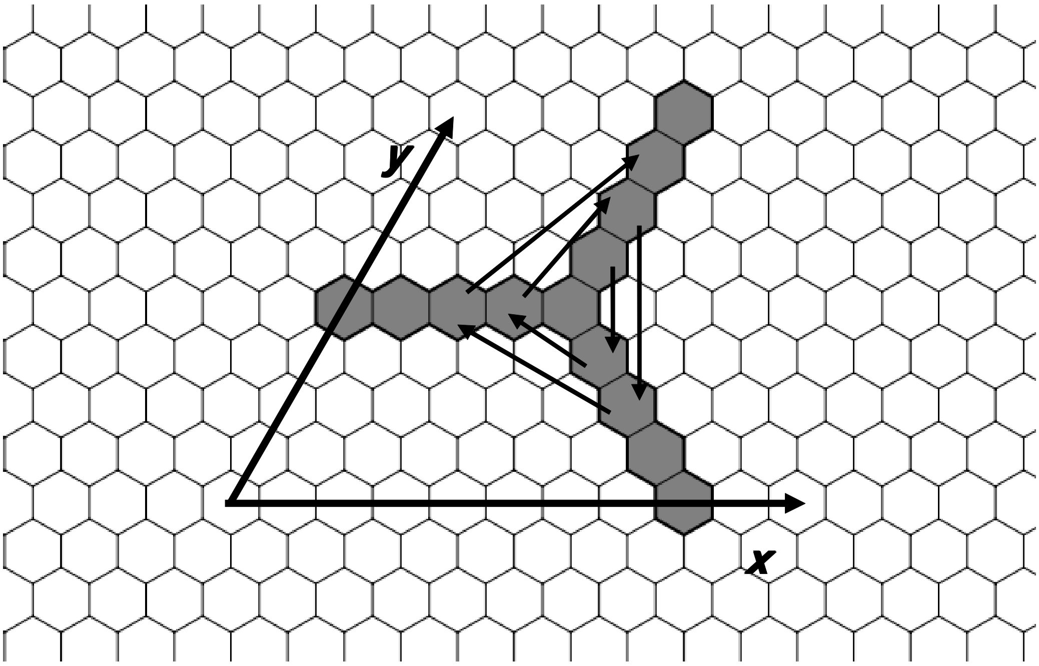

Figure 6. The procedure of adding hexagons to the benzenoid that have the

largest value of min(*X, Y, Z*)

Figure 7 shows that how the value of min(*X, Y, Z*) changes when hexagons are added one by one.

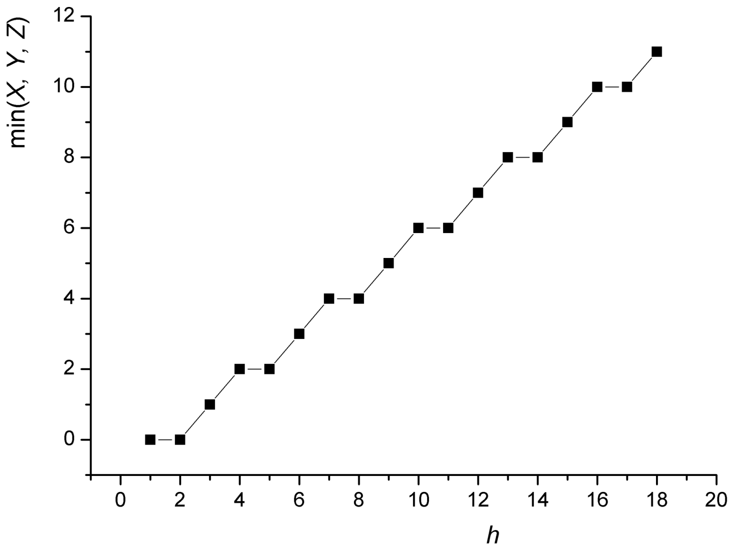

Figure 7. The values of min(*X, Y, Z*) as hexagons are added one by one

Thus, the equation to calculate min(*X, Y, Z*) is:

$$\min(\mathrm{X,Y,Z}) = \lfloor (h-1) \div 3 \rfloor \times 2 + a \quad \begin{cases} if\ (h-1) \bmod 3 = 2, & a = 1 \\ else, & a = 0 \end{cases}$$

The number of hexagons along the legs of the trapezoidal area is:

$$m = \min(X, Y, Z) + 1$$

$$m = \lfloor (h-1) \div 3 \rfloor \times 2 + 1 + a \quad \begin{cases} if\ (h-1) \bmod 3 = 2, & a = 1 \\ else, & a = 0 \end{cases}$$

The equation can be simplified as:

$$m = \left\lfloor \frac{2h+1}{3} \right\rfloor$$

It can be found in Figure 3 that if m=2, the upper base of trapezoid is h-1. Further,

it can be found that the length upper base is h-m+1.
