# Supplementary material for: Graph Theoretical Representation of Atomic Asymmetry and Molecular Chirality of Benzenoids in Two-Dimensional Space
Source: PLoS One. 2014 Jul 17;9(7):e102043. doi: 10.1371/journal.pone.0102043 (PMC4102468; doi:10.1371/journal.pone.0102043)
Supplement: File S2 — The isosceles trapezoid regions used to enumerate benzenoids composed of 3 to 11 benzene rings and all the benzenoids that have been enumerated. (ZIP) [file pone.0102043.s002.zip › Binary codes and position/region.pdf]

The isosceles trapezoid regions used to generate benzenoids composed of 3 to 11 benzene rings were listed as follows.

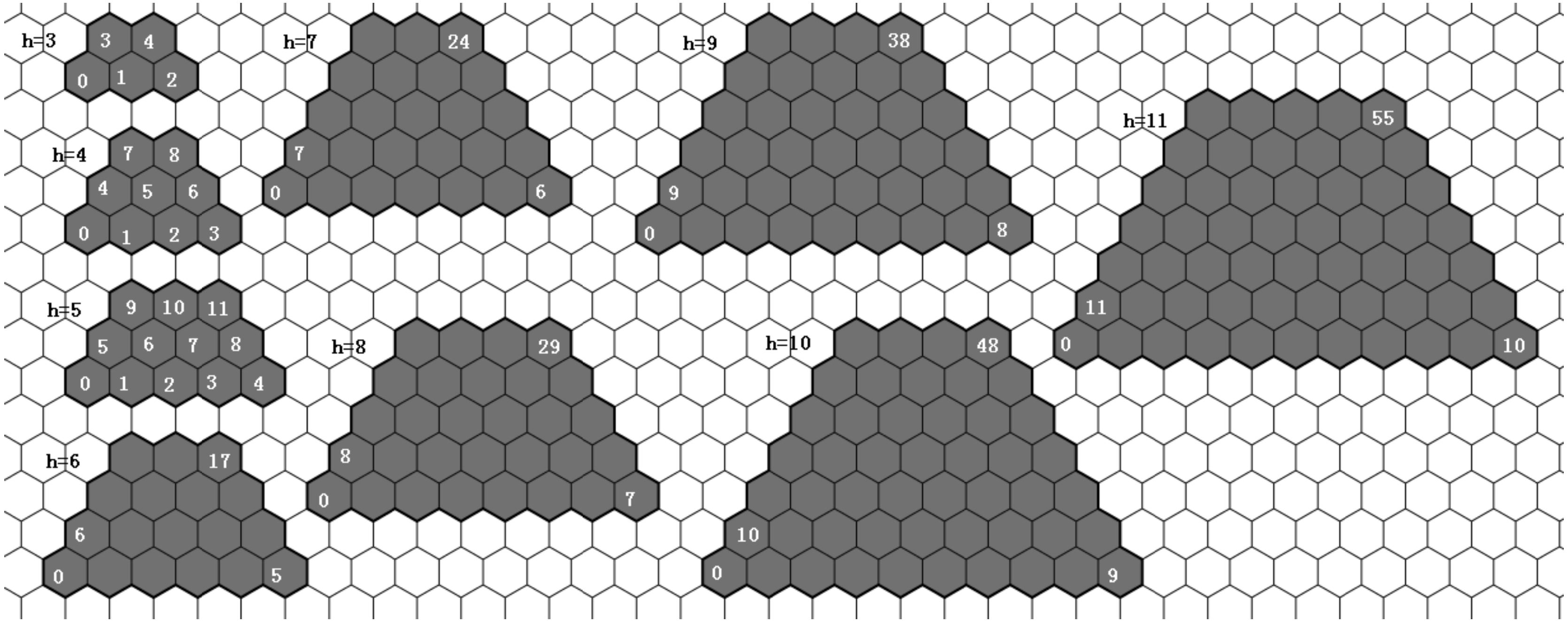

Each enumerated benzenoid can be represented by the combination of *h* hexagons in terms of hexagon numbers, which are listed in the two text files in supporting information.
